# *Bronchoscopy*

**DOI:** 10.1155/DTE.1.183

**Published:** 1995

**Authors:** J. Patrick Barron


					Diagnostic and Therapeutic Endoscopy, 1995, Vol. 1, p. 183
Reprints available directly from the publisher
Photocopying permitted by license only
(C) 1995 Harwood Academic Publishers GmbH
Printed in Malaysia
BOOK REVIEW
BRONCHOSCOPY, edited by Udaya B. S. Prakash, Raven Press, 1993, 560 pages, 862 illustrations (539 in color), 40 ta-
bles.
The title says it all--Bronchoscopy. This book is by far the most comprehensive work on the topic in the literature by
one of the leaders in the field of bronchoscopy Dr. Udaya B. S. Prakash of the Mayo Clinic.
Thetableofcontents gives an ideaoftheencyclopedic scopeofthisvolume,beginningwith theHistoryofBronchoseopy,
Anatomy for the Bronchoscopist, The Bronehoscopy Suite Equipment and Personnel, The Rigid Bronehoscope, The
Flexible Bronchoscope, Indications and Contraindieations to Bronehoscopy, Bronehoseopie Pharmacology and
Anesthesia, Preparation of the Patient for Bronehoscopy, Technical Solutions to Common Problems in Bronehoseopy,
Bronehoscopy in Peripheral and Central Lesions, Bronehoseopie Lung Biopsy, Bronehoscopie Needle Aspiration and
Biopsy, Bronchoalveolar Lavage, Bronehoscopy in Pulmonary Infections, Bronehoseopic Localization and Therapy of
Occult Lung Cancer, Therapeutic Bronchoscopy, Hemoptysis and Bronchoseopy-indueed Hemorrhage,Tracheobronchial
Foreign Bodies, Laser Bronehoscopy, Bronehoseopie Brachytherapy, Tracheobronehial Prosthesis, Bronehoscopie and
Thoracic Surgery, Pediatric Rigid Bronchoscopy, Pediatric Flexible Bronchoscopy, Complications of Bronchoseopy,
Processing of Bronchoscopy Specimens, Documentation ofBronchoseopie Findings, Maintenance of the Bronchoseope
and Bronchoscopy Equipment, Teaching Bronchoscopy, Optimal Bronehoscopy, Newer and Miscellaneous Applications
of Bronchoseopy and Atlas of Bronchoseopy.
Thirty experts from throughout the world havejoined with Dr. Prakash, who co-authored more than halfofthe 34 chap-
ters, in producing this definitive practical reference book that is aimed to meet the requirements of both beginning bron-
choscopists and experienced bronchoseopists of all levels. The style is eminently readable and should be easily
comprehensible even by readers whose first language is not English.
Each chapter is excellently organized and with extensive references making this book of unparalleled usefulness. All
bronehoseopists should ensure that they have rapid and convenient access to this remarkable work.
Prof. J. Patrick Barron
International Medical Communication Center
183

				

